# Large-Scale Survey of Intraspecific Fitness and Cell Morphology Variation in a Protoploid Yeast Species

**DOI:** 10.1534/g3.115.026682

**Published:** 2016-02-16

**Authors:** Paul P. Jung, Anastasie Sigwalt, Shinsuke Ohnuki, Jacky de Montigny, Yoshikazu Ohya, Joseph Schacherer

**Affiliations:** * Department of Genetics, Genomics and Microbiology, University of Strasbourg - Centre National de la Recherche Scientifique, Unité Mixte de Recherche 7156, 67083 Strasbourg, France; † Department of Integrated Biosciences, Graduate School of Frontier Sciences, University of Tokyo, Kashiwa 277-8562, Chiba, Japan

**Keywords:** phenotypes, fitness, cell morphology, genetic diversity, yeast

## Abstract

It is now clear that the exploration of the genetic and phenotypic diversity of nonmodel species greatly improves our knowledge in biology. In this context, we recently launched a population genomic analysis of the protoploid yeast *Lachancea kluyveri* (formerly *Saccharomyces kluyveri*), highlighting a broad genetic diversity (π = 17 × 10^−3^) compared to the yeast model organism, *S. cerevisiae* (π = 4 × 10^−3^). Here, we sought to generate a comprehensive view of the phenotypic diversity in this species. In total, 27 natural *L. kluyveri* isolates were subjected to trait profiling using the following independent approaches: (i) analyzing growth in 55 growth conditions and (ii) investigating 501 morphological changes at the cellular level. Despite higher genetic diversity, the fitness variance observed in *L. kluyveri* is lower than that in *S. cerevisiae*. However, morphological features show an opposite trend. In addition, there is no correlation between the origins (ecological or geographical) of the isolate and the phenotypic patterns, demonstrating that trait variation follows neither population history nor source environment in *L. kluyveri*. Finally, pairwise comparisons between growth rate correlation and genetic diversity show a clear decrease in phenotypic variability linked to genome variation increase, whereas no such a trend was identified for morphological changes. Overall, this study reveals for the first time the phenotypic diversity of a distantly related species to *S. cerevisiae*. Given its genetic properties, *L. kluyveri* might be useful in further linkage mapping analyses of complex traits, and could ultimately provide a better insight into the evolution of the genotype–phenotype relationship across yeast species.

Population genomic studies aim to explore both genetic and phenotypic diversity between different individuals belonging to the same species to obtain a better understanding of the mechanisms governing trait variation. Although genome diversity relies on different DNA variants that either affect only one DNA base pair or extend over many, phenotypic diversity may be investigated at various levels including the molecular (*e.g.*, transcriptome, proteome, metabolome), cellular (*e.g.*, cell morphology), fitness (*e.g.*, growth rate, yield of biomass, colony size), or visible features (*e.g.*, colony morphology, invasive growth, biofilm formation). The budding yeast *Saccharomyces cerevisiae* and other closely related species, including *S. paradoxus*, are the most often used for both genetic and phenotypic variation surveys (Schacherer *et al.* 2009; Liti *et al.* 2009; Warringer *et al.* 2011; Cromie *et al.* 2013; Bergstrom *et al.* 2014; Peter and Schacherer 2016). Whereas genomic data have exploded with the emergence of new generation sequencing technologies, phenotypic variation encounters the problem of throughput to investigate variation in a large number of traits across populations.

Significant progress has been made in recent years regarding the analysis of yeast growth, with the development of high-throughput phenotyping strategies based on semiautomated procedures such as the microcultivation approach (Warringer and Blomberg 2003; Jung *et al.* 2015) or colony size variation on solid media (Boone *et al.* 2007; Dittmar *et al.* 2010). Such advances have made possible the comparison of a large number of natural isolates in a reproducible and efficient manner. Large-scale studies have focused on various phenotypic traits including growth in response to different stress in both *S. cerevisiae* and *S. paradoxus*, as well as the fission yeast *Schizosaccharomyces pombe* (Kvitek *et al.* 2008; Warringer *et al.* 2011; Brown *et al.* 2011). Interestingly, it was shown that despite higher genetic diversity, phenotypic variation is much lower in *S. paradoxus* and *S. pombe* in comparison to *S. cerevisiae*, maybe because of the domestication of the latter, as fitness variation better reflects the population genetic history of a species than the adaptation to a specific ecological niche (Warringer *et al.* 2011).

Besides growth phenotypes, systematic and high-throughput exploration of quantitative morphological traits of yeast cells is also possible using an image-processing system, which automatically processes digital cell images (Ohya *et al.* 2015). Through deep investigation of deletion collection, almost half of the nonessential genes for growth have been shown to affect morphological traits in *S. cerevisiae* (Ohya *et al.* 2005). In addition, cell morphology variation across natural isolates of *S. cerevisiae* was also studied (Yvert *et al.* 2013; Skelly *et al.* 2013). In contrast to growth phenotypes, no impact of ecological or population genetic history on cell morphology variation was observed in *S. cerevisiae* (Yvert *et al.* 2013). Interestingly, the study of cell morphology provides a direct observation of individual cell behaviors in comparison to fitness, which reflects the contribution of cells living in a community (Ohya *et al.* 2015).

As mentioned above, the degree to which phenotypes vary across a species has mainly been investigated in both *S. cerevisiae* and *S. paradoxus* to date (Liti *et al.* 2009; Warringer *et al.* 2011). In order to establish major parallels between yeast and to observe behaviors specific to species, it is essential to explore the phenotypic variability in diverse genetic landscapes. In this context, we decided to explore the phenotypic diversity within the unexplored yeast species *Lachancea kluyveri* (formerly *S. kluyveri*). This latter is a heterothallic yeast having the same life cycle as that described for *S. cerevisiae*. It can live at both haploid and diploid levels; the two different mating types (*MAT*a and *MAT*α) can be crossed to form a stable diploid that can undergo meiosis under unfavorable conditions (McCullough and Herskowitz 1979). Thus, *L. kluyveri* can be suitable for classic as well as quantitative genetic studies.

This species is classified as protoploid, namely a species that did not undergo a Whole-Genome Duplication (WGD) event, in contrast to post-WGD species (Kellis *et al.* 2004). Genome sequencing and analysis revealed a 11.3 Mb genome spread on eight chromosomes (Genolevures *et al.* 2009). Recently, we sought to conduct a comprehensive polymorphism survey by sequencing both the mitochondrial and nuclear genomes of a large set of natural isolates (Jung *et al.* 2012; Friedrich *et al.* 2015). Nuclear genome evolution within this species showed a broad genetic diversity that can reach up to 2.5% and result in a nonstructured population. Moreover, this population genomic survey clearly demonstrated that distinct recombination and substitution regimes can coexist within a species and lead to different evolutionary patterns (Friedrich *et al.* 2015; Brion *et al.* 2015).

However, little is known about the phenotypic variation spectrum in *L. kluyveri*, despite some known differences when compared to *S. cerevisiae*, such as the inability to ferment sugars in the presence of oxygen (Moller *et al.* 2004) or the use of pyrimidines and its derivatives as a unique nitrogen source (Gojkovic *et al.* 1998; Beck *et al.* 2008). To gain a better overview of the phenotypic diversity within this species, we used high-throughput analyses based on growth fitness and cellular morphology. Fitness investigations relied on a microcultivation approach, where the growth of each strain was recorded under a large panel of 55 various growth conditions. In addition, intraspecific cellular morphology characterization was carried out using single-cell high-dimensional phenotyping based on microscopic images, where 501 morphological parameters were determined (Ohya *et al.* 2005). This large-scale analysis provides a first estimation of the phenotypic variation within a non-*Saccharomyces* species. Against all expectations, the growth variation was lower in *L. kluyveri* than in *S. cerevisiae* despite a higher genetic diversity, whereas an opposite trend was found for cellular morphology. Comparison of phenotypic patterns between isolates showed that trait variations (for fitness or morphological traits) follow neither the population history nor the source environment. However, fitness profiles are more similar between closely related strains than distant isolates. Altogether, our study provides multiple insights into the phenotypic diversity of *L. kluyveri*, which will be useful to future studies of natural genetic variation in this organism.

## Materials and Methods

### Strains and media

The strains used in this study were stable haploids or obtained from random spore analysis of natural diploid isolates. These strains were isolated from different geographical origins and ecological niches (Supplemental Material, Table S1). The media used to determine the phenotypes of each strain was the YPD medium (yeast extract 10 g/L, bactopeptone 20 g/L, dextrose 20 g/L) supplemented with various compounds (Table S2). In the “Carbon Utilization” class, dextrose was substituted by alternative carbon sources (Table S2). The minimal medium YNB (yeast nitrogen base 6.7 g/L, dextrose 20 g/L) was also used during this analysis.

### Morphological trait analysis

Cell morphological data of 27 *L. kluyveri* strains were acquired according to the methods described previously (Yvert *et al.* 2013). Briefly, yeast cells were grown in synthetic growth medium (SD; yeast nitrogen base without amino acids 6.7 g/L, glucose 20 g/L). Cells were cultured in 20 ml of liquid SD medium at 30°C to logarithmic-phase. Cell fixation, staining, and image acquisition were performed as described previously (Ohya *et al.* 2005). At least 200 cells were captured in a set of acquired images from an independent cell culture. A total of 135 sets of images were acquired from five replicate experiments on each of the 27 strains. The image sets were processed with CalMorph software (version 1.3) as described previously (Yvert *et al.* 2013).

### High-throughput phenotyping using a microcultivation approach

Strains were subjected to high-throughput phenotyping by a microcultivation strategy in biological duplicate (n = 2). Briefly, strains were inoculated in 150 μl of YPD medium and incubated overnight at 30°. Precultures were picked in 96-well microplates containing 150 μl of different media (Table S2) and cultivated for 48 hr in a microplate reader (Tecan Infinite F200) at 30°C unless otherwise specified. These plates were stirred for 8 min with orbital shaking (120 rpm) followed by 2 min with linear shaking (160 rpm) over the course of 48 hr. After each stirring cycle, absorbance was recorded using a narrow spectrum (595–605 nm). In each tested condition, we determined the specific growth rate (population doubling time), the lag time (population adaptation time), and the efficiency (biomass produced during the cultivation) using a “home-made” program. Briefly, after background correction, the specific growth rate was determined given the maximum linear regression line calculated over 15 consecutive absorbance records with a sliding window of 10 points. Lag time was estimated as the intersection between this regression line and the baseline absorbance level. The yield of biomass was determined as the maximal OD reached during the cultivation process. Each value was normalized using results obtained with standard YPD medium, representing 1485 data points for each of the three parameters.

### Data analysis

All statistical analyses of the cell morphology were performed using the R programming language (http://www.r-project.org/). The Kruskal-Wallis test, a principal component analysis (PCA), and a hierarchical cluster analysis (HCA) were performed as described previously (Yvert *et al.* 2013). First, 15 PCs accounting for 90% of the cumulative contribution ratio were used for HCA (Figure S1). Each PC was characterized in Table S3 using the morphological features by grouping the morphological traits as described previously (Ohnuki *et al.* 2014). Phenotypic variance of the cell morphology among 27 strains was calculated from the Z values of 501 traits, which were estimated by the generalized linear model (GLM) as described previously (Yang *et al.* 2014). Phenotypic variance among growth rates was estimated as the average of the variance estimated in each environmental condition.

### Data availability

The authors state that all data necessary for confirming the conclusions presented in the article are represented fully within the article.

## Results

### Fitness variation within a natural population of L. kluyveri

The ability to grow and the estimation of doubling time in specific environmental conditions (*e.g.*, different carbon sources or temperatures) are common parameters allowing for the characterization of the variation between and within species, particularly in yeasts. To obtain insight concerning intraspecific variation within the *L. kluyveri* species, we determined the specific growth rate, lag time, and the yield of biomass produced during the cultivation process of 27 *L. kluyveri* strains (listed in Table S1) grown in 55 environmental conditions, which were classified into three main groups: carbon utilization, toxins, and environments and metabolites (Table S2). With the exception of the strain 55-86.1, which was a strain prone to the flocculation phenotype, the same set of strains as those used for unraveling the genetic architecture within *L. kluyveri* were used (Friedrich *et al.* 2015). Overall the reproducibility between biological duplicate is high (Pearson correlation R = 0.95) suggesting low experimental variation. In YPD conditions, the specific growth rates ranged from 0.18 hr^−1^ for the strain 68917.2 to 0.72 hr^−1^ for the strain NRBC1811. For the reference strain CBS3082a, the doubling time is ∼1.15 hr, which is similar to that of *S. cerevisiae*.

As described in both *S. cerevisiae* and *S. paradoxus*, a strong correlation was found when comparing the growth rate and the biomass yield (Pearson correlation R = 0.78) in most of the experimental conditions tested (with the exception of the substitution of glucose by mannitol or ethanol showing a null correlation). In contrast, the lag phase was independent from these fitness features (R=−0.11 and −0.08 for growth rate and yield of biomass, respectively) (Figure S2) (Warringer *et al.* 2011). Together, these data suggest that a common genetic architecture governs both growth rate and biomass formation. To gain a better overview of the impact of the different environmental conditions on fitness, the growth of every strain in each condition was characterized and normalized to the standard YPD medium (see *Materials and Methods*).

**Figure 1:**
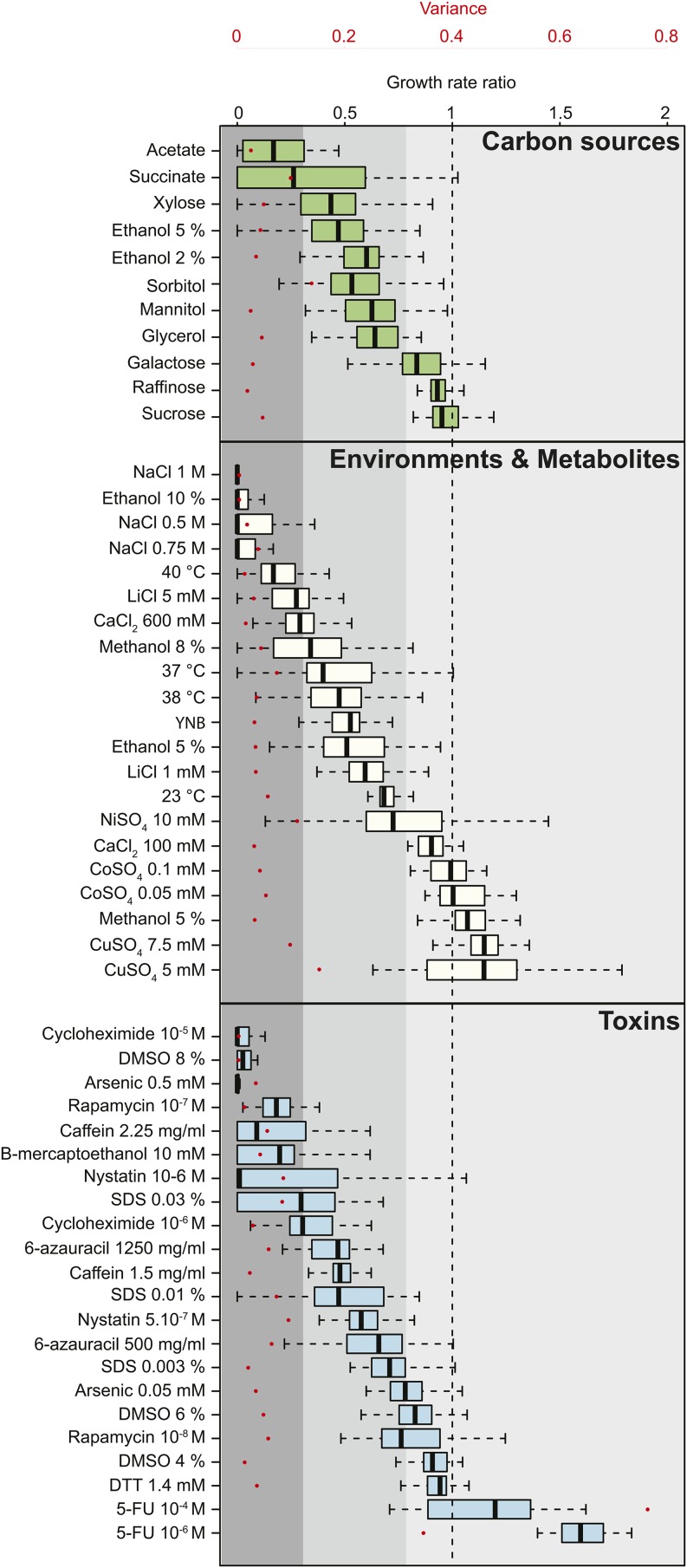
Intraspecific variance of fitness. Growth rates were determined in each condition and normalized to YPD (yeast extract 10 g/L, bactopeptone 20 g/L, dextrose 20 g/L) for the 27 strains. A ratio of 1 (dotted line) represents no variation in comparison to YPD. Growth conditions were classified as carbon sources (green), environment and metabolites (ivory), or toxins (blue). The variance among all strains in each condition is depicted by red dots corresponding to the secondary axis. Conditions were ranked according to their median calculated for the 27 strains. Three groups of strains were determined depending on the resistance: low (median < 0.3, dark gray), mid (median 0.3–0.8, intermediate gray), and high resistance (median > 0.8, light gray).

Barring xylose, growth on fermentable sugars generated little difference in growth compared to glucose. In contrast, nonfermentable sources led to a decrease in the specific growth rates, perhaps due to different uptake mechanisms and metabolization of these sugars (Figure 1). Similarly, three subgroups of isolates were found for the environments and metabolites and toxins classes: one corresponded to very low growth reflecting high sensitivity, as it is the case for conditions that alter osmolarity; one corresponded to weak growth such as in the case where temperature was altered; and the last subgroup corresponded to growth similar to that observed in YPD (Figure 1). Strikingly, the 5-FU metabolite generated an enhanced growth rate at concentrations that *S. cerevisiae* strains are sensitive to (Jaquet *et al.* 1993), probably due to the ability of *L. kluyveri* to use pyrimidine nucleotides and derivatives as a nitrogen source (Andersen *et al.* 2006).

The estimated variance for both of the 5-FU concentrations was at least twice as high as in the other tested conditions, suggesting that these isolates are characterized by a very broad resistance to this metabolite. Generally, the variance within the different conditions was low but some growth environments led to higher variation, such as succinate or the presence of metal ions (*e.g.*, NiSO_4_ or CuSO_4_) (Figure 1). This suggests that mechanisms of resistance to specific substrates are variable between different strains. The low variance estimated in the standard YPD condition (0.013) may reflect a very similar growth profile among all isolates in this condition. However, strain 68.917.2 is a clear outlier as it explains almost 50% of the total variance. Indeed, by removing the growth rate data of 68.917.2, the variance decreased from 0.013 to 0.008. Over all, these data suggest that intraspecies evolution allows for differential resistance to various stresses among some strains.

### Cell morphology variation within a natural population of L. kluyveri

**Figure 2:**
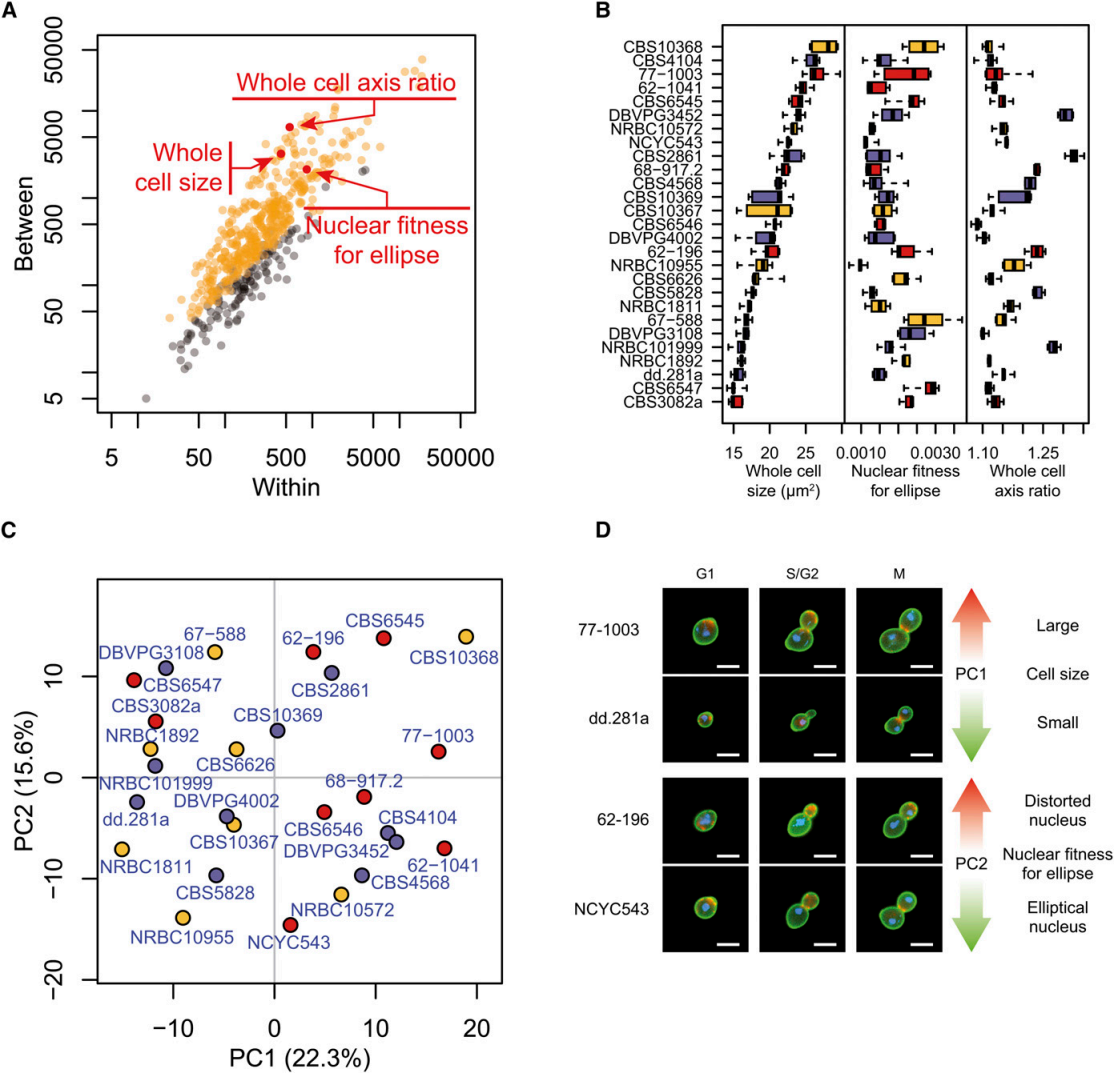
Morphological parameter variation within the *L. kluyveri* species. (A) Intra- *vs.* interstrain variability of morphological traits. Each dot represents one of 501 measured parameters. Orange and gray distinguish the traits that were called significant and nonsignificant by the Kruskal-Wallis test at *P* < 0.05 after Bonferroni correction, respectively. For the purpose of visual clarity, each parameter was transformed by f(x) = (x − μCBS10367) / σCBS10367, where μCBS10367 and σCBS10367 are the mean and standard deviation of the parameter across five replicates of the CBS10367 strain. Note that significance inference was determined from ranks of raw values and was therefore not affected by this transformation. The sum of squares across replicates (x-axis) and across strains (y-axis) was then computed. The three parameters highlighted in red reflect distinct cellular properties: whole cell size of the G1 cell, nuclear fitness for ellipse of the S/G2 cell, and long over short axis ratio of the ellipse fitted to the G1 cell. (B) Boxplot representation of these morphological parameters calculated for the 27 *L. kluyveri* strains. Box colors represent geographical origins with red for America, blue for Europe, and yellow for Asia. (C) Principal component (PC) analysis of *L. kluyveri* morphological variation. Strains are represented by their coordinates along the first two principal components, using the same colors as in Figure 2B. (D) Representative cells illustrating the traits contributing to the first two principal components. Bar: 5 μm.

Analysis of morphology includes a set of phenotypic traits among which variation was previously characterized in the budding *S. cerevisiae* yeast (Yvert *et al.* 2013). We performed high-dimensional morphological phenotyping of the same 27 *L. kluyveri* isolates after cultivation in complete medium at 30°. These strains predominantly contained unattached individual cells rather than aggregates of unseparated cells, making it possible to perform semiautomated image analysis with CalMorph (Ohya *et al.* 2005). Briefly, each strain was cultivated in five biological replicates. Cells were then fixed with formaldehyde and their cell wall, nuclear DNA, and actin were stained with various specific fluorescent dyes. Images of at least 200 cells per culture were acquired via fluorescent microscopy and analyzed with CalMorph software to quantify 501 traits reflecting the size, shape, orientation, and intracellular organization of the cells. Despite a low variance of the growth rate determined in complete medium, 384 morphological traits revealed significant intraspecies variability out of the total 501 traits tested (*P* < 0.05 after Bonferroni correction by Kruskal-Wallis test, Figure 2A). Detecting a great number of differences (77%) across the 27 strains suggested that the majority of the morphological organization in *L. kluyveri* is subjected to intraspecies quantitative variation.

One of the most striking variations in morphology concerned whole cell size (Figure 2A). In Figure 2B, the *L. kluyveri* strains are aligned in descending order of cell size with CBS10368 characterized by the largest cell size, approximately 1.7 times larger than that of the smallest strain, CBS3082a. Two additional traits that dramatically varied across strains were related to the nuclear shape (nuclear fitness for ellipse) and the elongated cell shape (mother axis ratio). The values of these three traits ranked strains in three different orders, indicating that there was no correlation among cell size, nuclear shape, and elongated cell shape. Thus, the variation of *L. kluyveri* cellular morphology represents a set of multiple independent traits with different sources of variability.

### Trait variation and population history

**Figure 3:**
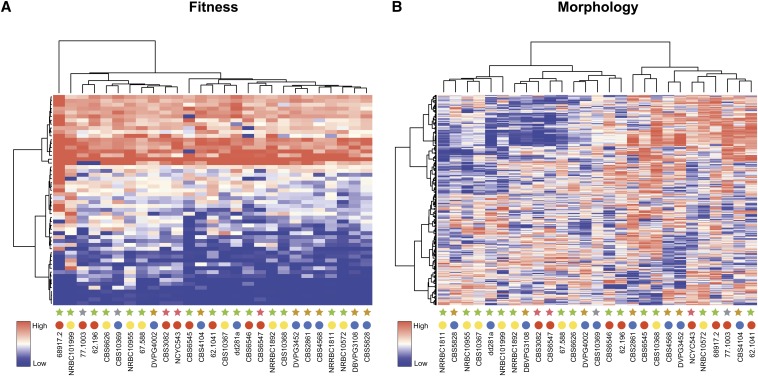
Trait variation within *L. kluyveri* is not related to geographical or ecological niches. Hierarchical clustering of trait profiles was performed using a centered Pearson correlation metric and average linkage mapping for growth (A) as well as morphological parameter (B) analyses. In both heatmaps, rows and columns correspond to conditions (or morphological parameters) and strains, respectively. Growth rates were normalized for each condition to YPD (yeast extract 10 g/L, bactopeptone 20 g/L, dextrose 20 g/L); the high growth rates are depicted in red and slow growth rates in blue in the color scale. Similarly, in the heatmap corresponding to morphological data, low to high morphological score values are depicted by a gradient from blue to red. Colored circles represent geographical origins of each strain: Europe (blue), America (red), and Asia (yellow). Similarly, stars depict ecological origins of each strain: tree exudate and leaf (green), soil (brown), insect gut (purple), and unknown (gray).

The genetic divergence within the *L. kluyveri* species ranges up to 2.5% (Friedrich *et al.* 2015), whereas trait profiling and morphological feature characterization show little variation. Hierarchical clustering of fitness data obtained for the 27 natural isolates of *L. kluyveri* reveal two main clusters that are not correlated with either their ecological or geographical origins (Figure 3A). Except for the strains isolated from Europe that are mainly found in group 2 (7 out of 9 strains), the strains are evenly distributed between clusters. It is notable that strain 68.917.2 does not belong to either of the two groups, and appears to be an outlier within the *L. kluyveri* species, probably due to its slow growth on YPD. Hierarchical clustering carried out with the different classes of environmental conditions analyzed here identified similar groups clearly, suggesting that trait evolution is independent of strain niche according to the conditions tested (Figure S3).

Similarly, hierarchical clustering of the 501 morphological parameters revealed two clusters, each divided into two subgroups where no correlation with strain origin was distinguishable (Figure 3B). We then investigated the properties of the variation in morphological features variation using principal component analysis (PCA). The first two components were correlated with cell size and nuclear fitness for ellipse, respectively (Figure 2C and Table S3). We found that the strains were nearly evenly spaced with no particular subgroups apparent. Analysis of all spaces represented by the first five components (63% of the cumulative contribution ratio, Figure S1) led to similar results (Figure S4), suggesting that *L. kluyveri* is characterized by a continuum of morphological features rather than discrete classes of distinct morphologies. In accordance with hierarchical clustering, strains from common ecological origins did not group together, indicating that differences in the morphological traits do not simply reflect adaptation to the simulated environments.

### Relationship between genetic and phenotypic diversity

**Figure 4:**
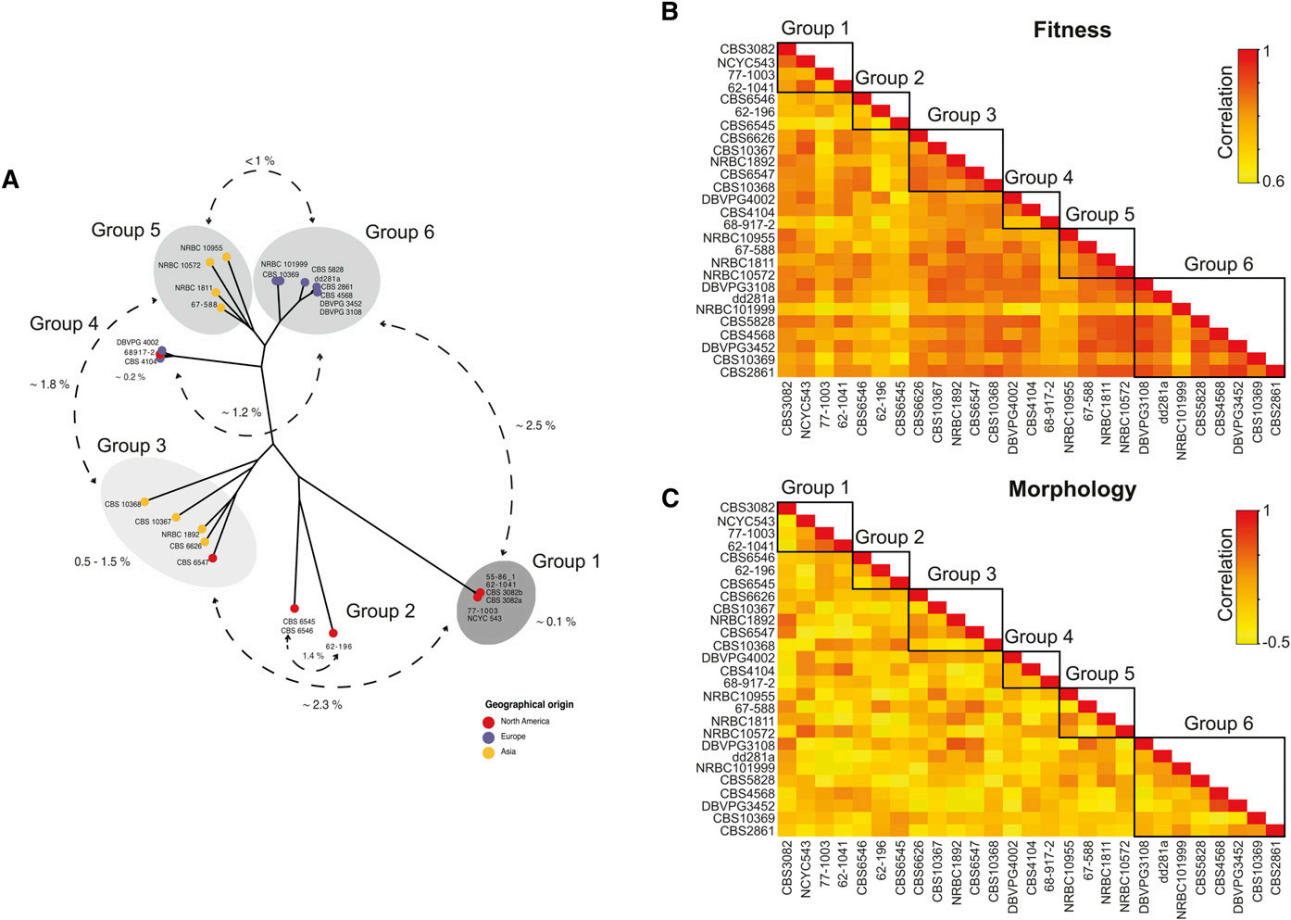
Correlation between and within specific groups of *L. kluyveri*. (A) Genetic diversity within the *L. kluyveri* based on more than 881,427 segregating sites leads to the distinction of 6 different subgroups. Colored circles represent geographical origins of each strain: Europe (blue), America (red), and Asia (yellow). Pairwise comparison for growth rate (B) and morphological parameters (C) revealed high correlation to fitness independent of the subgroups, whereas no clear correlation was found for morphological data. The color scale represents levels of correlation where high correlation is depicted in red and low correlation in yellow.

According to 881,427 polymorphic sites determined previously within the *L. kluyveri* species (Friedrich *et al.* 2015), genetic architecture led to the definition of six subgroups, containing the 27 strains used in this study (Figure 4A). Among these subgroups, there were two clearly defined populations: one corresponded to the strains that had originated from America (group 1) and the second to those isolated from Europe (group 6) (Figure 4A) (Friedrich *et al.* 2015). The average pairwise comparison of trait profiles within the *L. kluyveri* species gave a correlation of 0.8. This parameter appears to be variable within and between the different groups. Indeed, the estimated correlation in the group 6 is approximately 0.85 whereas it decreases to 0.7 in group 2 (Figure 4B). In contrast, comparison of morphological parameters revealed an average correlation of nearly 0 (−0.034), due to the significant differences among the 384 cellular features. In spite of this almost null correlation, we were also able to determine variation between and within the different groups, for example a weak anticorrelation was estimated (R= −0.2) between groups 3 and 4 (Figure 4C).

**Figure 5:**
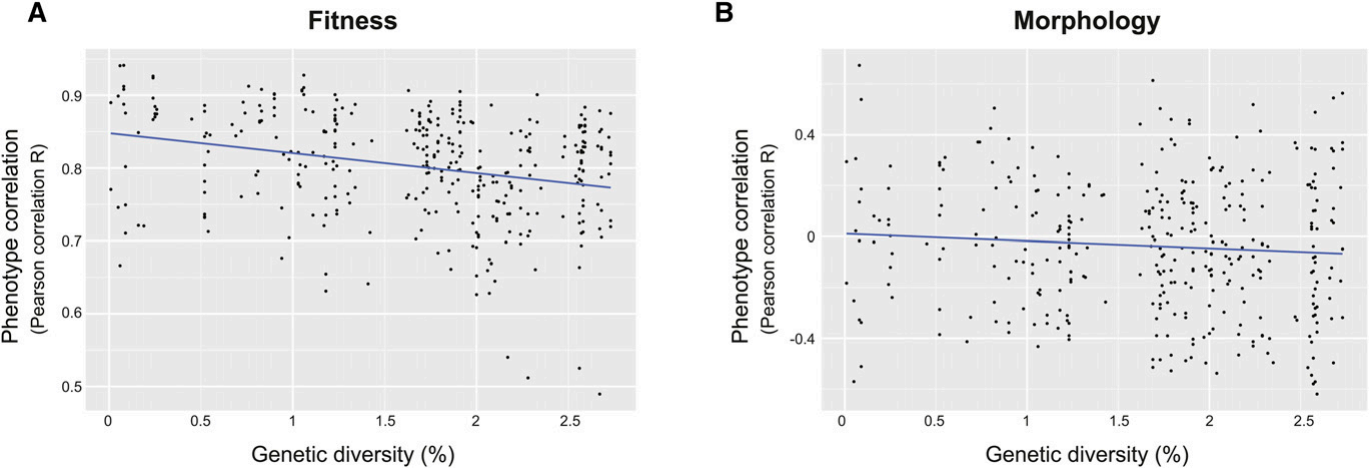
Correlation between genetic diversity and trait profile variation. Pairwise genetic diversity is plotted against pairwise Pearson correlation calculated for both fitness (A) and morphological parameters (B). The blue line corresponds to linear regressions.

To better understand how genetic variation drives phenotypic variability, we determined the relationship between genetic diversity and trait profiles. Strains belonging to group 1 were characterized by less similar trait profiles (average correlation of 0.8) despite their high genetic conservation. However, a significant anticorrelation (Kendall’s test, *P*-value = 3.94 × 10^−9^) was found when comparing fitness data among all the 27 strains, indicating that trait variation increases with the enhancement of genetic diversity (Figure 5A). In contrast, no such correlation was determined between morphological features and genetic variation (Kendall’s test, *P*-value = 0.296) (Figure 5B). These results suggest that genetic diversity within *L. kluyveri* influences trait profiles, but cannot explain all of the phenotypic variability given the lower correlation among North American strains.

### Comparison of trait variation across different yeast species

**Figure 6:**
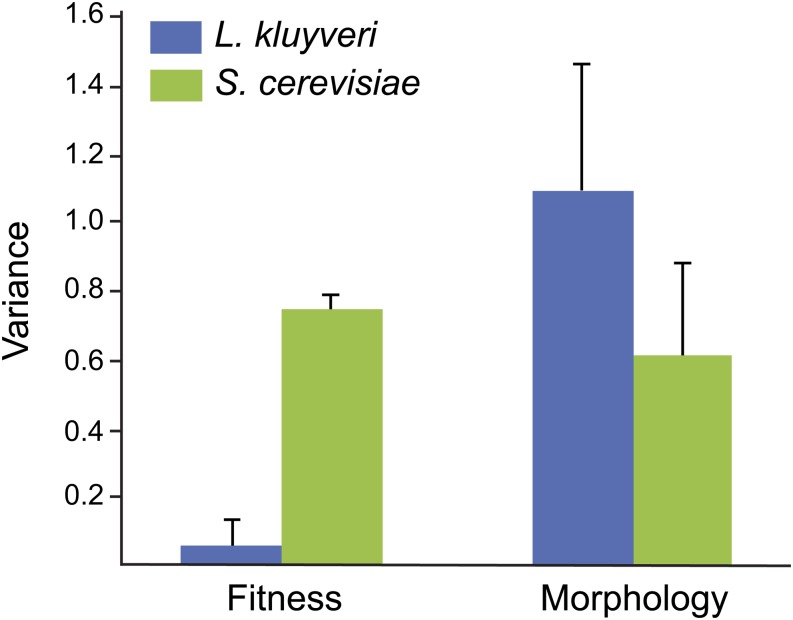
Fitness and morphological parameters variance. Comparison of phenotypic variance within *L. kluyveri* (blue) and *S. cerevisiae* (green) for growth data and morphological parameters.

Trait variation analysis within *S. cerevisiae* revealed a correlation with their genomic structure rather than to adaptation associated with their ecological niche. For example, *S. cerevisiae* strains isolated from Europe are more prone to be resistant to Na^+^ and Li^+^ ions given the pleiotropic roles of causal loci. In addition, clean lineages within the *S. paradoxus* species show similar trait profiles (Warringer *et al.* 2011). As mentioned above, we previously identified two clean lineages within the *L. kluyveri* species: one corresponding to strains originated from North America (group 1), the second comprised of strains isolated from Europe (group 6) (Friedrich *et al.* 2015). In contrast to *S. cerevisiae*, trait profile and morphological feature analyses revealed variation independently from the genetic structure of the *L. kluyveri* species. Moreover, the evolution of resistance to various stresses including heavy metals or high osmolarity is not related to strain niche. The lack of correlation between growth phenotype or cellular parameters on one hand, and the population structure or the origins of the strains on the other, could be related to the small number of isolates available from *L. kluyveri*. Despite this low number of strains, the overall genetic diversity is greater than 2.5%, whereas it is ∼0.5% for *S. cerevisiae*. In sharp contrast, the phenotypic variance is lower in *L. kluyveri* than in *S. cerevisiae* (Figure 6), which has also been described for both *S. paradoxus* and *S. pombe* species (Warringer *et al.* 2011; Brown *et al.* 2011). However, morphological variance determined in this study for *L. kluyveri* shows a clear trend where it is much higher than in *S. cerevisiae* (Figure 6) (Yvert *et al.* 2013). Altogether, these data suggest that cellular morphology and growth are clearly two independent features given the impact of genetic diversity on their variation. Nevertheless, the small number of strains analyzed could lead to biased results and a larger collection would be required to definitively characterize morphological variation.

## Discussion

Assessing the diversity among individuals within a species is now feasible for nonmodel organisms and essential for understanding population history, as well as obtaining a better insight into the genotype–phenotype relationship (Peter and Schacherer 2016). The Saccharomycotina subphylum (budding yeasts), which includes the baker’s yeast *S. cerevisiae*, includes ideal nonmodel organisms with which to explore diversity in terms of genetic and phenotypic variation (Freel *et al.* 2014, 2015, 2016). In this study, we investigated phenotypic variation within the protoploid *L. kluyveri* species by analyzing variation in both growth and morphological parameters, allowing for a comparison with previous studies performed in *S. cerevisiae* (Yvert *et al.* 2013; Warringer *et al.* 2011). Interestingly, the general behavior of these two categories of phenotype is very different within as well as between species.

Among the 501 morphological features investigated in logarithmically growing cells, 384 showed significant variation among the strains studied, suggesting that these traits are subjected to large intraspecific variation within *L. kluyveri*. Similarly, 440 morphological parameters were previously described as significantly variable among 37 strains of *S. cerevisiae* (Yvert *et al.* 2013). Comparison of cell morphology between *L. kluyveri* and *S. cerevisiae* allowed us to clearly distinguish these two species according to the size of the nuclei or the buds, for instance. *L. kluyveri* has an extremely high variance in cell morphology of 1.1 compared to 0.62 determined with previously published data generated in *S. cerevisiae* (Yvert *et al.* 2013). In contrast, growth analysis of the same set of *L. kluyveri* isolates in 55 different environmental conditions showed a very low variance (0.06), demonstrating that growth profiles are more similar than that for *S. cerevisiae* species, where the variance was estimated to be approximately 0.75 (Warringer *et al.* 2011).

In contrast to fitness patterns in *S. cerevisiae*, trait variation (for fitness and cell morphology) is not defined by population history in *L. kluyveri*. Using PCA analysis and hierarchical clustering, we did not observe any correlation between the origin (ecological or geographical) and the phenotypic patterns, showing that trait variations are not linked to either the population structure or the source environment. However, the number of *L. kluyveri* strains used in this study is relatively low and their ecological origins are somehow similar. Increasing the number of strains would probably reveal more structured trait profiles.

Finally, we determined the correlation between pairwise trait variation and genetic diversity in order to have a better view of how genomes and phenotypes are related. Interestingly, there is strong evidence that genetic diversity is anticorrelated with fitness variation. However, this is not the case for differences among morphological parameters, showing a different global behavior of the two types of traits. We are here considering genetic diversity as SNPs but other genome variation can lead to trait profile differences. Genomes of the 27 strains of *L. kluyveri* were obtained through mapping against a reference genome (Friedrich *et al.* 2015). Although this method gives deep information on genome variation, important features are still missing such as transposon content, structural variants, or the presence of specific genes for a distinct group of strains (pan genome). Moreover, prion proteins can also serve as a motor for phenotypic inheritance in *S. cerevisiae* but are still not well known in *L. kluyveri* (Halfmann *et al.* 2012). A better knowledge of these features would help to gain a better understanding of phenotypic diversity.

Given the genetic characteristics of *L. kluyveri* and its trait variability, quantitative genetics is definitively applicable to this species and would lead to a better understanding of the genotype–phenotype relationship across diverse yeasts. Moreover, laboratory evolution experiments carried out for both *S. cerevisiae* and the pathogenic *Candida albicans* species have shown that the presence of an additional chromosome is linked to stress resistance acquisition (Selmecki *et al.* 2009; Yona *et al.* 2012). Similar experiments in *L. kluyveri* would reveal if the acquisition of aneuploidy is a common mechanism across yeast species to improve fitness in stress conditions. Finally, further analysis will, without a doubt, provide deeper insight into functional diversity in this nonmodel organism.

## 

## Supplementary Material

Supplemental Material
