# Supplementary material for: Large-Scale Survey of Intraspecific Fitness and Cell Morphology Variation in a Protoploid Yeast Species
Source: G3 (Bethesda). 2016 Feb 16;6(4):1063–71. doi: 10.1534/g3.115.026682 (PMC4825641; doi:10.1534/g3.115.026682)
Supplement: Supplemental Material [file supp_6_4_1063__index.html]

Large-Scale Survey of Intraspecific Fitness and Cell Morphology Variation in a Protoploid Yeast Species — Supplemental Material 

# Large-Scale Survey of Intraspecific Fitness and Cell Morphology Variation in a Protoploid Yeast Species

## Supplemental Material for Jung *et al.*, 2016

**Files in this Data Supplement:**

- Supporting Materials - File contains Figures S1-S4, Tables S1-S2, and the legend for Table S3. (.pdf, 1,005 KB)
- Figure S1 - Cumulative proportion of variance of the principal component analysis for the phenotypes. (.pdf, 35 KB)
- Figure S2 - Relationships between specific growth rate, lag phase and yield of biomass. (.pdf, 456 KB)
- Figure S3 - Trait variation with *L. kluyveri* according to the environmental condition classes. (.pdf, 63 KB)
- Figure S4 - Pair plots of PC scores for the 27 *L. kluyveri* species. (.pdf, 97 KB)
- Table S1 - List of strains used in this study. (.pdf, 102 KB)
- Table S2 - List of tested conditions. (.pdf, 131 KB)
- Table S3 - Principal component loadings used for the characterization of each PC. (.xlsx, 17 KB)
