# Supplementary material for: Large-Scale Survey of Intraspecific Fitness and Cell Morphology Variation in a Protoploid Yeast Species
Source: G3 (Bethesda). 2016 Feb 16;6(4):1063–71. doi: 10.1534/g3.115.026682 (PMC4825641; doi:10.1534/g3.115.026682)
Supplement: Supplemental Material [file supp_g3.115.026682_026682SM.pdf]

Supporting Materials

Large-scale survey of the intraspecific fitness and cell morphology variation in a protoploid yeast species

Paul P. Jung, Anastasie Sigwalt, Shinsuke Ohnuki, Jacky de Montigny, Yoshikazu Ohya, and Joseph Schacherer

Supporting Figures

Figure S1. Cumulative proportion of variance of the principal component analysis for the phenotypes. Grey bars indicate the proportion of variance (left axis) explained by the PCs. Red circles indicate the cumulative proportion of variance (right axis) explained by the PCs. The horizontal dashed red line indicates 60% and 90% of the cumulative proportion of variance.

Figure S2. Relationships between specific growth rate, lag phase and yield of biomass. The fitness parameters were extracted from high-density growth curves. A high correlation is found when comparing growth rates and yield of biomass (A) whereas the lag phase feature do not correlate with neither yield of biomass (B) nor growth rates (C).

Figure S3. Trait variation with *L. kluyveri* according to the environmental condition classes. Hierarchical clustering of trait profiles was performed using a centered Pearson correlation metric and average linkage mapping for carbon sources, toxins and environment and metabolites classes. The color scale represents results where high growth rates are depicted in red and slow growth rates in blue.

Figure S4. Pair plots of PC scores for the 27 *L. kluyveri* species. Principal component analysis of *L. kluyveri* morphological variation. Strains are represented by their coordinates along the first five principal components. Box colors represent geographical origins as red with America, purple with Europe, and orange with Asia, using the same colors as in Figure 2B.

Supporting Tables

Table S1. List of strains used in this study.
Table S2. List of tested conditions.
Table S3. Principal component loadings used for the characterization of each PC.

Figure S1

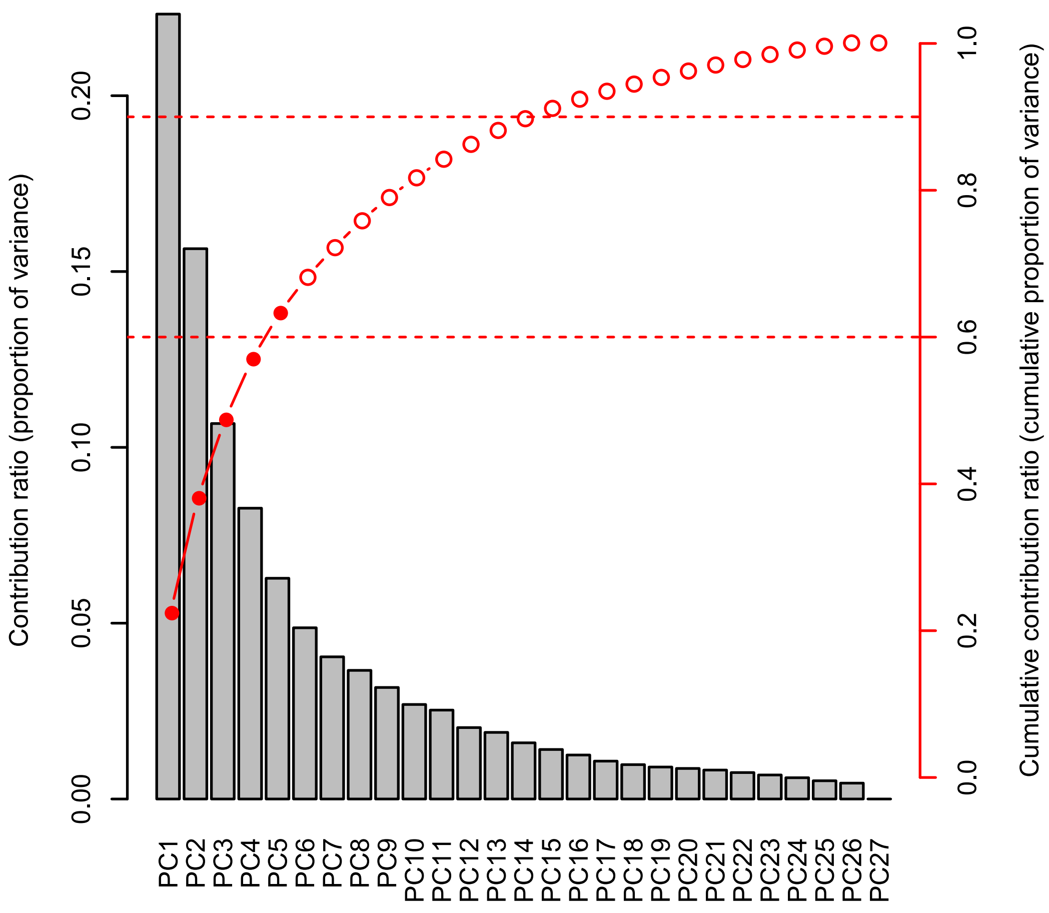

Contribution ratio (proportion of variance)
0.00
0.05
0.10
0.15
0.20
Cumulative contribution ratio (cumulative proportion of variance)
0.0
0.2
0.4
0.6
0.8
1.0
PC1
PC2
PC3
PC4
PC5
PC6
PC7
PC8
PC9
PC10
PC11
PC12
PC13
PC14
PC15
PC16
PC17
PC18
PC19
PC20
PC21
PC22
PC23
PC24
PC25
PC26
PC27

Figure S2

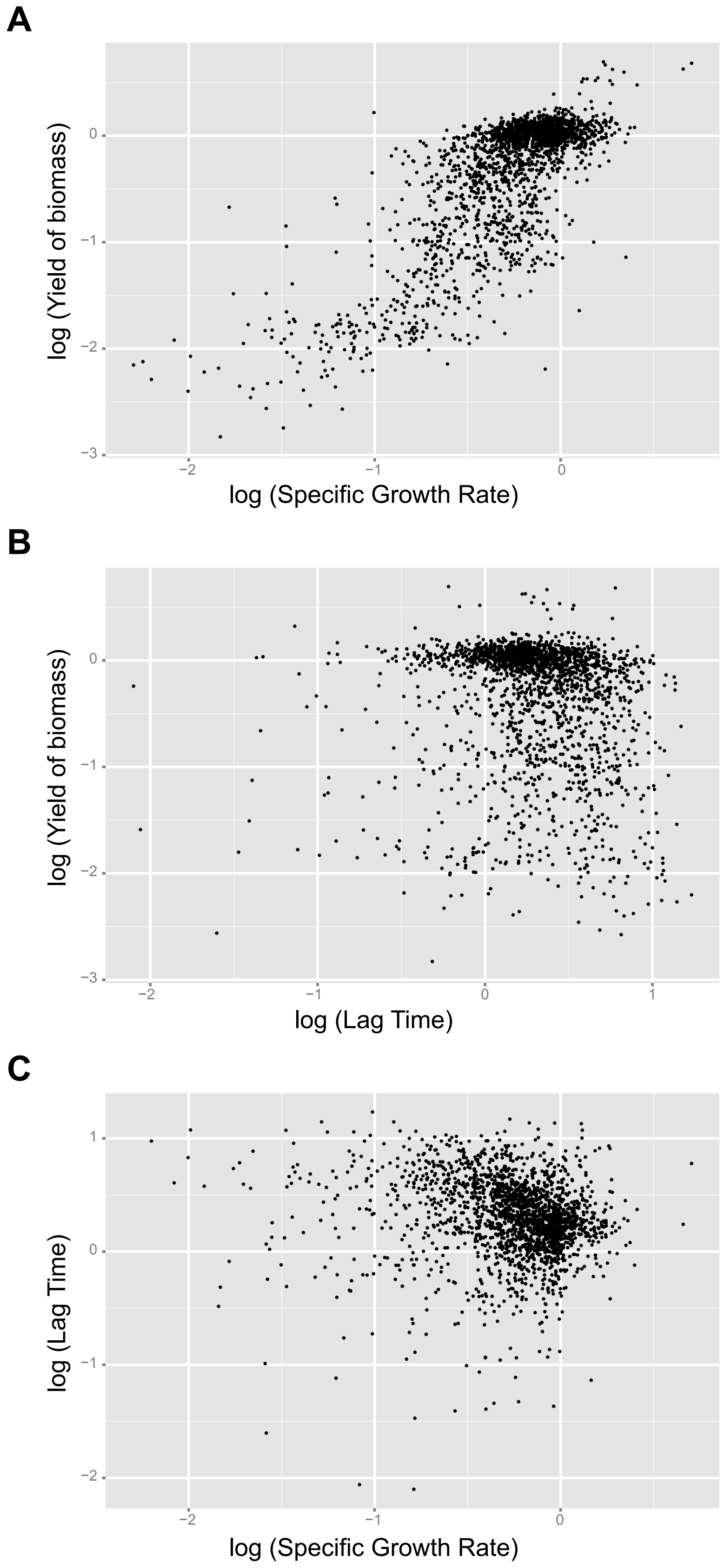

A
log (Yield of biomass)
0
−1
−2
−3
−2
−1
0
log (Specific Growth Rate)
B
log (Yield of biomass)
0
−1
−2
−3
−2
−1
0
1
log (Lag Time)
C
log (Lag Time)
1
0
−1
−2
−2
−1
0
log (Specific Growth Rate)

Figure S3

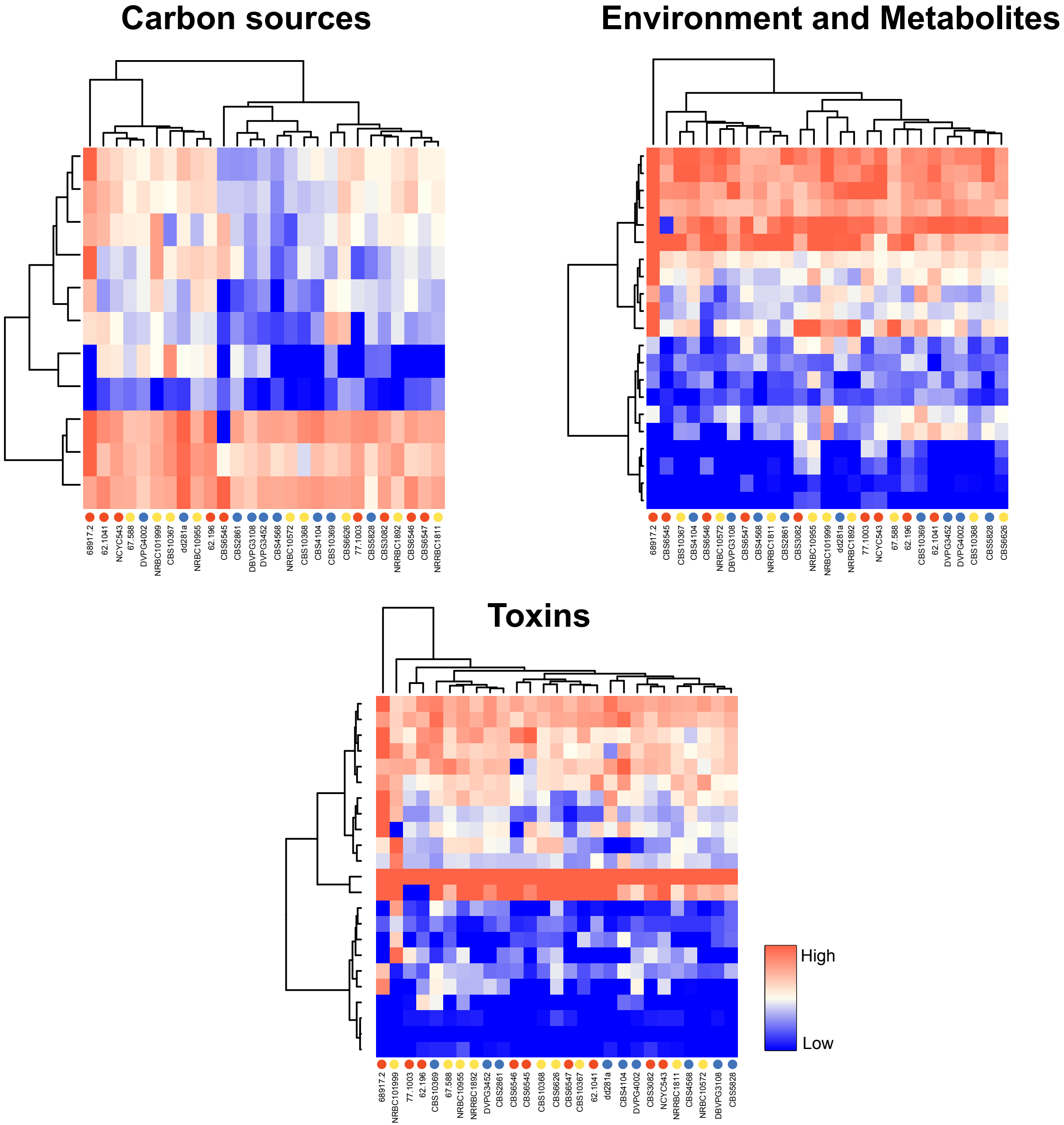
Carbon sources
68917.2
62.1041
NCYC543
67.588
DVPG4002
NRBC101999
CBS10367
dd281a
NRBC10955
62.196
CBS6545
CBS2861
DBVPG3108
DVPG3452
CBS4568
NRBC10572
CBS10368
CBS4104
CBS10369
CBS6626
77.1003
CBS5828
CBS3082
NRRBC1892
CBS6546
CBS6547
NRRBC1811
Environment and Metabolites
68917.2
CBS6545
CBS10367
CBS4104
CBS6546
NRBC10572
DBVPG3108
CBS6547
CBS4568
NRRBC1811
CBS2861
CBS3082
NRBC10955
NRBC101999
dd281a
NRRBC1892
77.1003
NCYC543
67.588
62.196
CBS10369
62.1041
DVPG3452
DVPG4002
CBS10368
CBS5828
CBS6626
Toxins
68917.2
NRBC101999
77.1003
62.196
CBS10369
67.588
NRBC10955
NRRBC1892
DVPG3452
CBS2861
CBS6546
CBS6545
CBS10368
CBS6626
CBS6547
CBS10367
62.1041
dd281a
CBS4104
DVPG4002
CBS3082
NCYC543
NRRBC1811
CBS4568
NRBC10572
DBVPG3108
CBS5828
High
Low

Figure S4

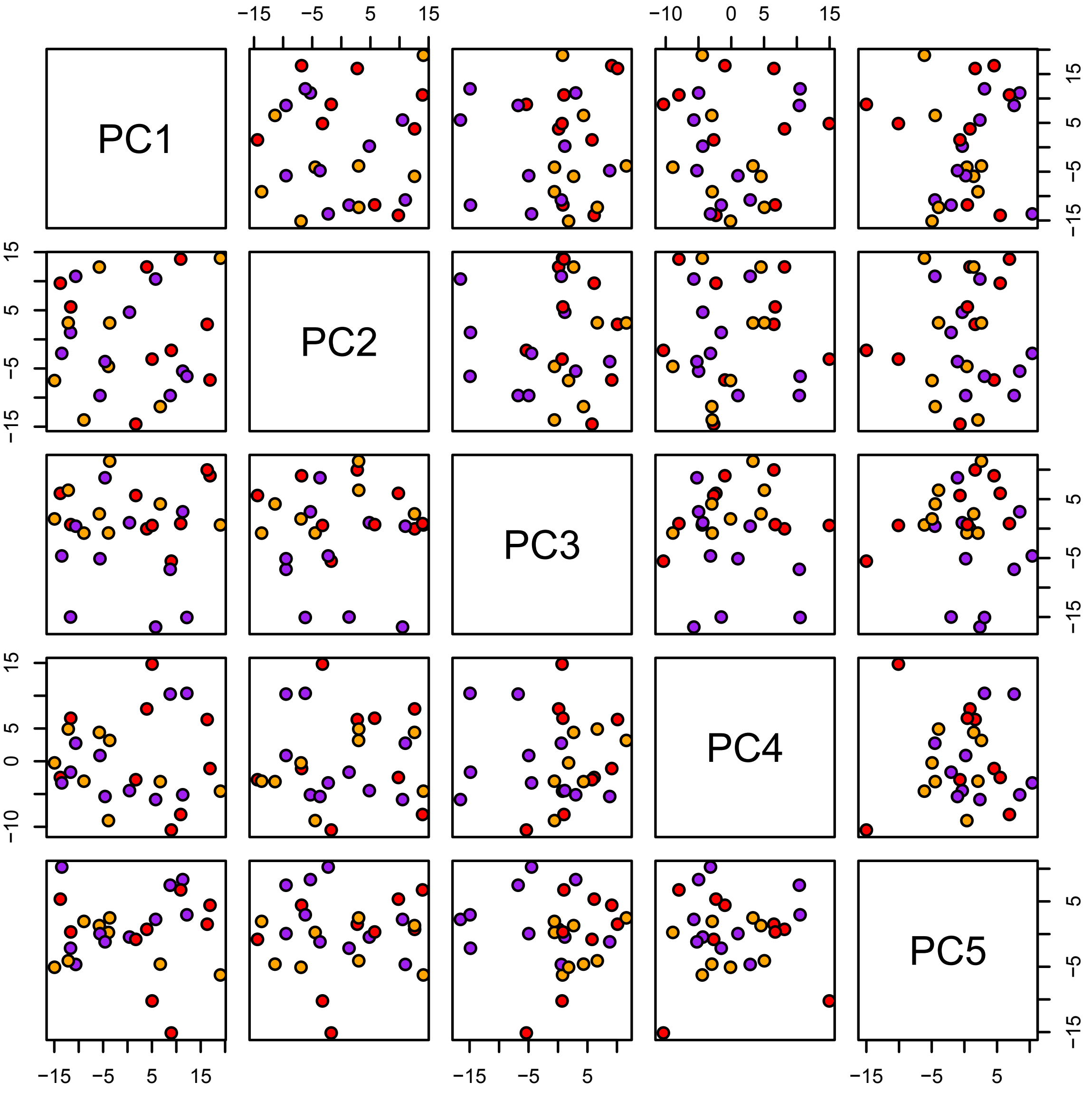

Table S1. Strains used in this study

Strain	Ecological niches	Geographical origins
**62-1041**	Willow exudate	USA, California, Davis
**CBS 3082**	*Drosophila pinicola*	USA, California
**77-1003**	Unknown	USA, California
**NCYC 543**	*Drosophila pinicola*	USA, California
**62-196**	*Taraxacum* officinale	Canada, Saskatoon
**CBS 6545**	Salix exudate	USA, California
**CBS 6546**	Exudate of Populus sp.	USA, California
**CBS 6547**	*Drosophila pseudobscura*	USA, California, Gualala Creek
**CBS 6626**	Exudate of tree	Japan, Mt. Takamatsu
**NRBC 1892**	Tree exudate	Japan
**CBS 10367**	*Quercus Mongolica* exudate	Russia, Kedrova pas natural reserve
**CBS 10368**	*Quercus Mongolica* exudate	Russia, Sikhote-Alinsky nature reserve
**CBS 4104**	Soil	Netherlands, Wageningen
**68.917-2**	Exudate of Populus trichocarpa	Canada
**DBVPG 4002**	Cavern	Italy
**67-588**	*Ulmus japonica* exudate	Japan, Yamabe
**NRBC 1811**	Partially decaying leaf	Japan
**NRBC 10572**	Moss	Thailland
**NRBC 10955**	Decaying leaf of Rhizophora mucronata	Japan
**CBS 10369**	Unknown	Spain
**NRBC 101999**	Decaying leaf of Rhizophora mucronata	Japan
**CBS 5828**	Soil	Denmark
**dd281a**	Forest	Germany, Heidelberg
**CBS 2861**	Soil	Sweden
**CBS 4568**	Soil	Sweden
**DBVPG 3452**	Soil	Sweden, Ultana
**DBVPG 3108**	Soil	Netherlands

Table S2. Conditions used in this study. The classification “Carbon utilization” indicates that the 2% of dextrose was substituted by other carbon sources.

Condition	Concentration	Class
**Acetate**	2 %	Carbon source
**Ethanol**	2 %	Carbon source
**Ethanol**	5 %	Carbon source
**Galactose**	2 %	Carbon source
**Glucose**	2 %	Carbon source
**Glycerol**	2 %	Carbon source
**Mannitol**	2 %	Carbon source
**Raffinose**	2 %	Carbon source
**Sorbitol**	2 %	Carbon source
**Succinate**	2 %	Carbon source
**Sucrose**	2 %	Carbon source
**Xylose**	2 %	Carbon source
**$CaCl_2$**	600 mM	Environment
**$CaCl_2$**	100 mM	Environment
**Cold**	23 °C	Environment
**$CoSO_4$**	0.1 mM	Environment
**$CoSO_4$**	0.05 mM	Environment
**$CuSO_4$**	7.5 mM	Environment
**$CuSO_4$**	5 mM	Environment
**Ethanol**	10 %	Environment
**Ethanol**	5 %	Environment
**Heat**	40 °C	Environment
**Heat**	37 °C	Environment
**Heat**	38 °C	Environment
**LiCl**	5 mM	Environment
**LiCl**	1 mM	Environment
**Methanol**	8 %	Environment
**Methanol**	5 %	Environment
**NaCl**	1 M	Environment
**NaCl**	0.5 M	Environment
**NaCl**	0.75 M	Environment
**NiSO4**	10 mM	Environment
**YNB**		Environment
**5-FU**	10^{-4} M	Toxins
**5-FU**	10^{-6} M	Toxins
**6-azauracil**	1250 mg/ml	Toxins
**6-azauracil**	500 mg/ml	Toxins
**Arsenic**	0.5 mM	Toxins
**Arsenic**	0.05 mM	Toxins
**B-**	10 mM	Toxins

**mercaptoethanol**		
**Caffein**	2.25 mg/ml	Toxins
**Caffein**	1.5 mg/ml	Toxins
**Cycloheximide**	10^{-5} M	Toxins
**Cycloheximide**	10^{-6} M	Toxins
**DMSO**	8 %	Toxins
**DMSO**	6 %	Toxins
**DMSO**	4 %	Toxins
**DTT**	1.4 mM	Toxins
**Nystatin**	10^{-6} M	Toxins
**Nystatin**	5.10^{-7} M	Toxins
**Rapamycin**	10^{-7} M	Toxins
**Rapamycin**	10^{-8} M	Toxins
**SDS**	0.03 %	Toxins
**SDS**	0.01 %	Toxins
**SDS**	0.003 %	Toxins
