# Supplementary figures and images for: Large-Scale Survey of Intraspecific Fitness and Cell Morphology Variation in a Protoploid Yeast Species
Source: G3 (Bethesda). 2016 Feb 16;6(4):1063–71. doi: 10.1534/g3.115.026682 (PMC4825641; doi:10.1534/g3.115.026682)

**Figure S1**

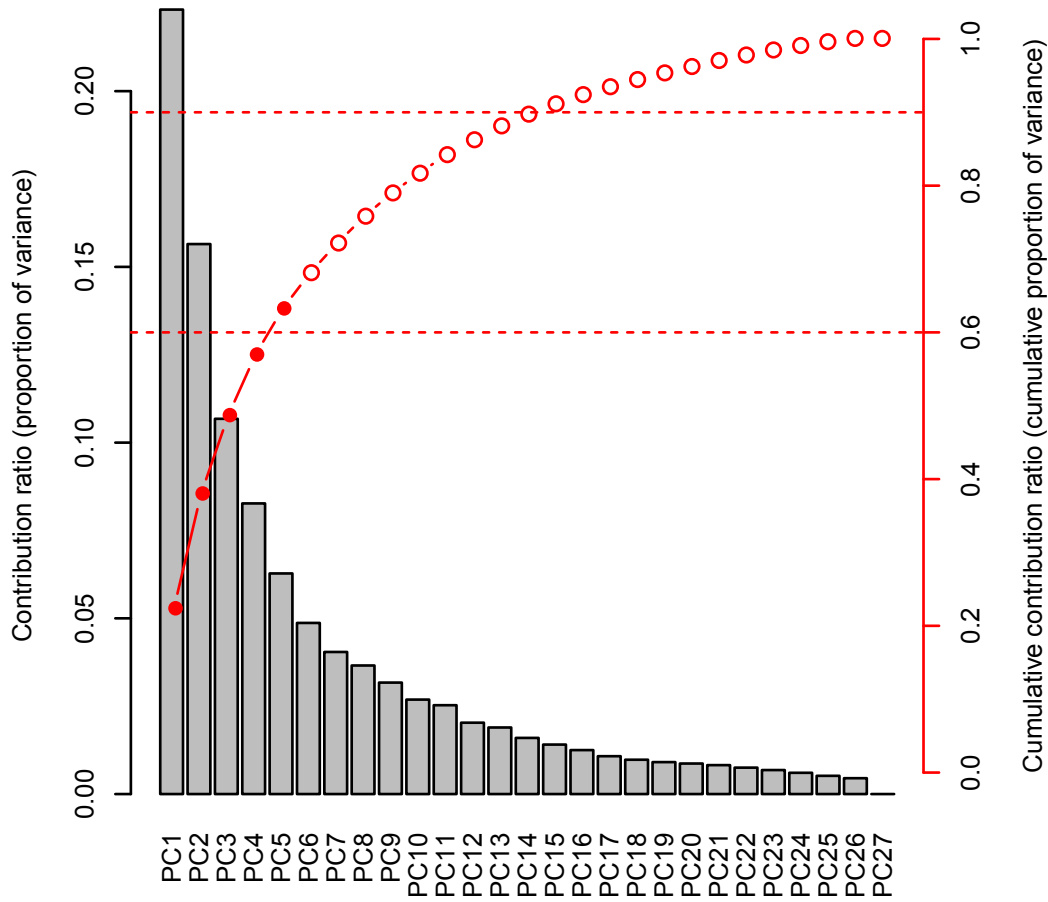

Supplement: Supplemental Material [file supp_g3.115.026682_FigureS1.pdf]

Figure S2

A

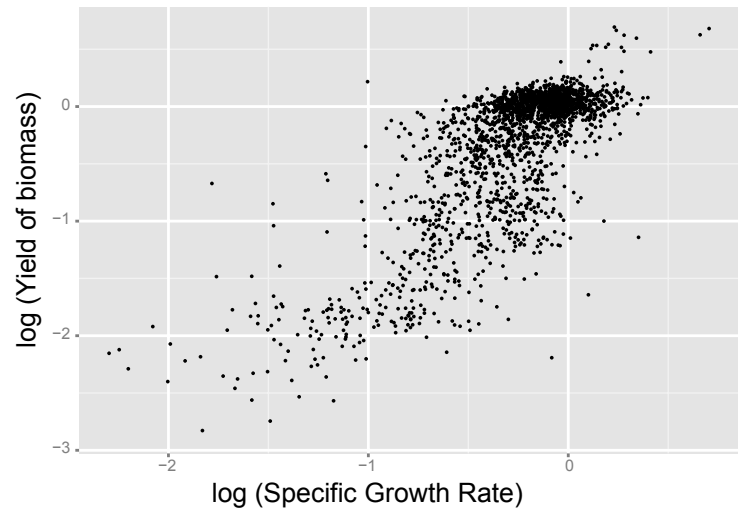

B

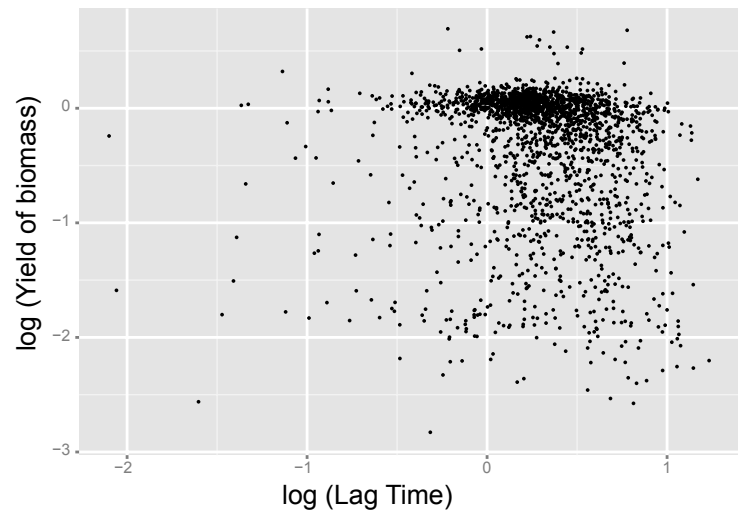

C

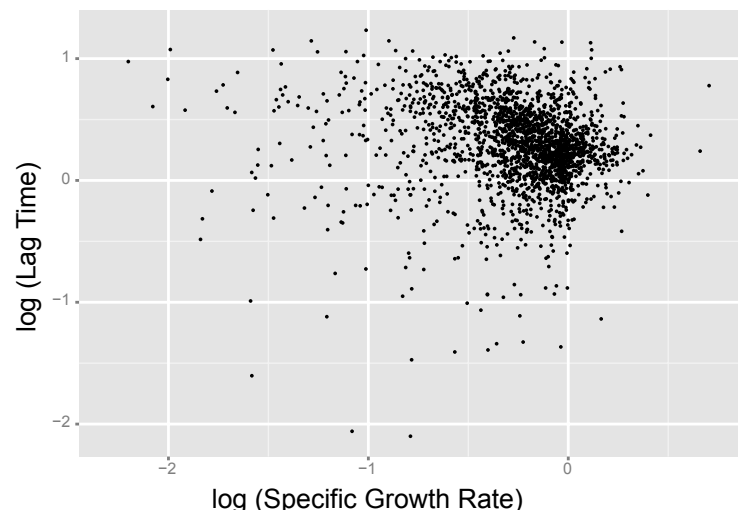

Supplement: Supplemental Material [file supp_g3.115.026682_FigureS2.pdf]

Figure S3

Carbon sources

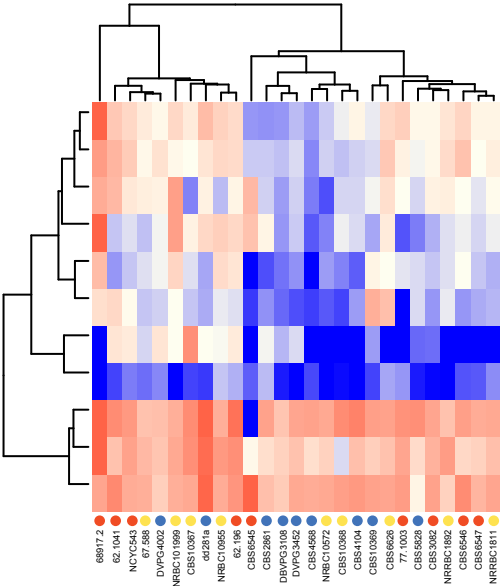

Environment and Metabolites

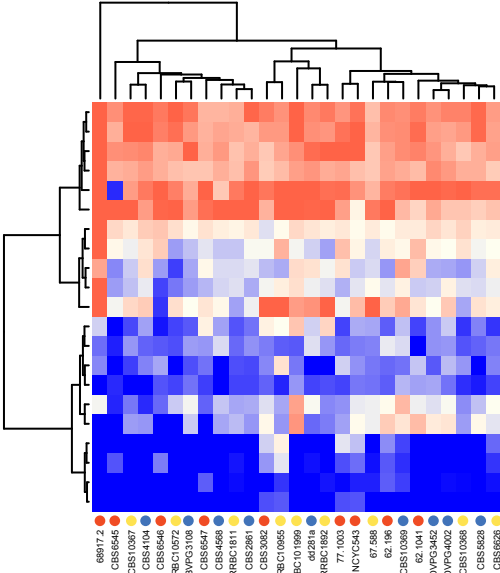

Toxins

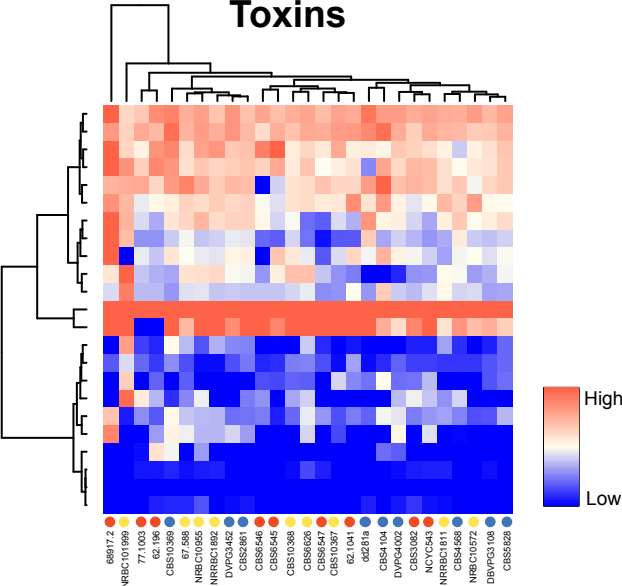

Supplement: Supplemental Material [file supp_g3.115.026682_FigureS3.pdf]

**Figure S4**

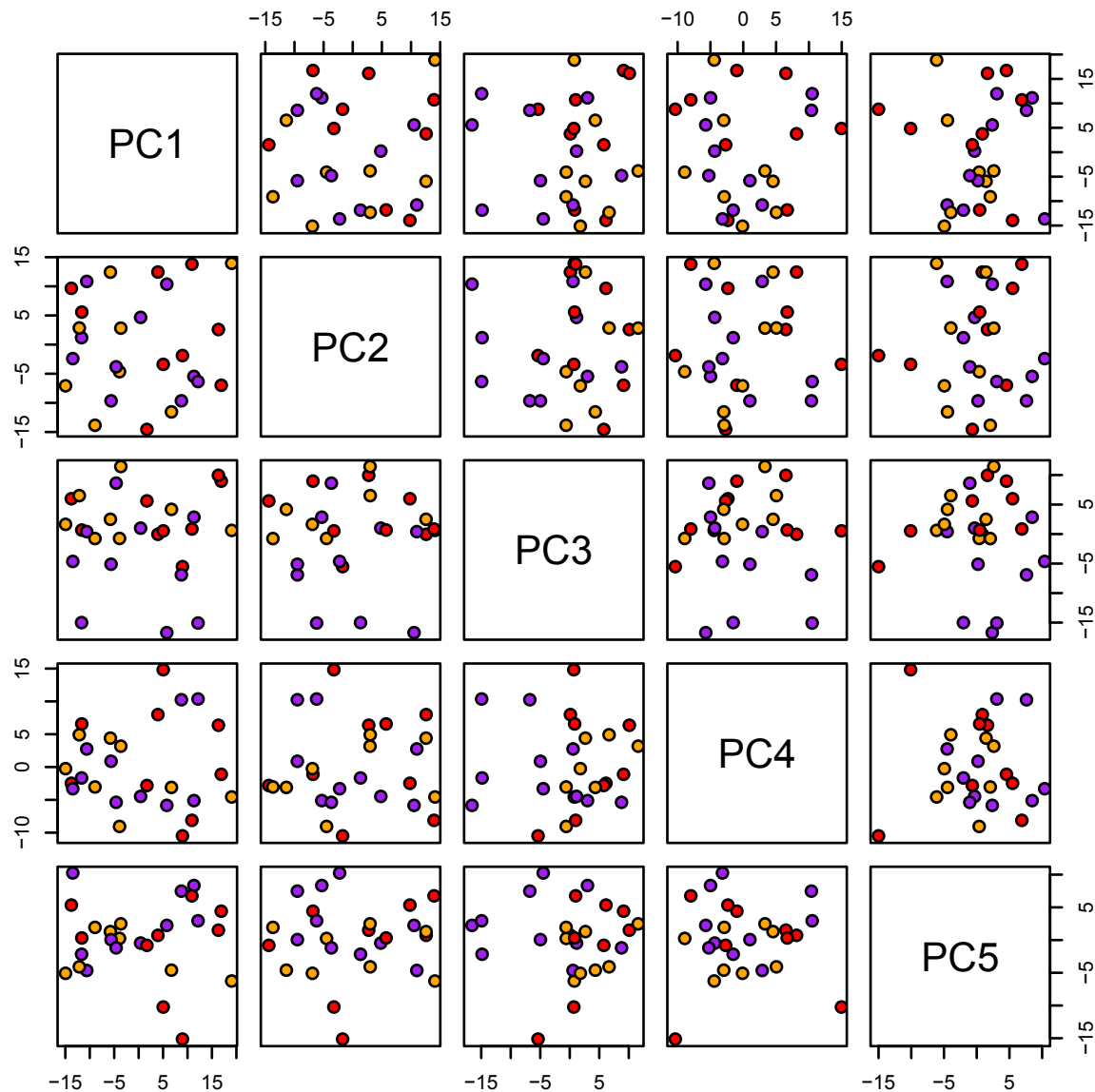

Supplement: Supplemental Material [file supp_g3.115.026682_FigureS4.pdf]
