# Supplementary material for: Large-Scale Survey of Intraspecific Fitness and Cell Morphology Variation in a Protoploid Yeast Species
Source: G3 (Bethesda). 2016 Feb 16;6(4):1063–71. doi: 10.1534/g3.115.026682 (PMC4825641; doi:10.1534/g3.115.026682)
Supplement: Supplemental Material [file supp_g3.115.026682_TableS1.pdf]

Table S1. Strains used in this study

Strain	Ecological niches	Geographical origins
**62-1041**	Willow exudate	USA, California, Davis
**CBS 3082**	*Drosophila pinicola*	USA, California
**77-1003**	Unknown	USA, California
**NCYC 543**	*Drosophila pinicola*	USA, California
**62-196**	*Taraxacum* officinale	Canada, Saskatoon
**CBS 6545**	Salix exudate	USA, California
**CBS 6546**	Exudate of Populus sp.	USA, California
**CBS 6547**	*Drosophila pseudobscura*	USA, California, Gualala Creek
**CBS 6626**	Exudate of tree	Japan, Mt. Takamatsu
**NRBC 1892**	Tree exudate	Japan
**CBS 10367**	*Quercus Mongolica* exudate	Russia, Kedrova pas natural reserve
**CBS 10368**	*Quercus Mongolica* exudate	Russia, Sikhote-Alinsky nature reserve
**CBS 4104**	Soil	Netherlands, Wageningen
**68.917-2**	Exudate of Populus trichocarpa	Canada
**DBVPG 4002**	Cavern	Italy
**67-588**	*Ulmus japonica* exudate	Japan, Yamabe
**NRBC 1811**	Partially decaying leaf	Japan
**NRBC 10572**	Moss	Thailland
**NRBC 10955**	Decaying leaf of Rhizophora mucronata	Japan
**CBS 10369**	Unknown	Spain
**NRBC 101999**	Decaying leaf of Rhizophora mucronata	Japan
**CBS 5828**	Soil	Denmark
**dd281a**	Forest	Germany, Heidelberg
**CBS 2861**	Soil	Sweden
**CBS 4568**	Soil	Sweden
**DBVPG 3452**	Soil	Sweden, Ultana
**DBVPG 3108**	Soil	Netherlands
