# Supplementary material for: Large-Scale Survey of Intraspecific Fitness and Cell Morphology Variation in a Protoploid Yeast Species
Source: G3 (Bethesda). 2016 Feb 16;6(4):1063–71. doi: 10.1534/g3.115.026682 (PMC4825641; doi:10.1534/g3.115.026682)
Supplement: Supplemental Material [file supp_g3.115.026682_TableS2.pdf]

Table S2. Conditions used in this study. The classification "Carbon utilization" indicates that the 2% of dextrose was substituted by other carbon sources.

Condition	Concentration	Class
**Acetate**	2 %	Carbon source
**Ethanol**	2 %	Carbon source
**Ethanol**	5 %	Carbon source
**Galactose**	2 %	Carbon source
**Glucose**	2 %	Carbon source
**Glycerol**	2 %	Carbon source
**Mannitol**	2 %	Carbon source
**Raffinose**	2 %	Carbon source
**Sorbitol**	2 %	Carbon source
**Succinate**	2 %	Carbon source
**Sucrose**	2 %	Carbon source
**Xylose**	2 %	Carbon source
**$CaCl_2$**	600 mM	Environment
**$CaCl_2$**	100 mM	Environment
**Cold**	23 °C	Environment
**$CoSO_4$**	0.1 mM	Environment
**$CoSO_4$**	0.05 mM	Environment
**$CuSO_4$**	7.5 mM	Environment
**$CuSO_4$**	5 mM	Environment
**Ethanol**	10 %	Environment
**Ethanol**	5 %	Environment
**Heat**	40 °C	Environment
**Heat**	37 °C	Environment
**Heat**	38 °C	Environment
**LiCl**	5 mM	Environment
**LiCl**	1 mM	Environment
**Methanol**	8 %	Environment
**Methanol**	5 %	Environment
**NaCl**	1 M	Environment
**NaCl**	0.5 M	Environment
**NaCl**	0.75 M	Environment
**NiSO4**	10 mM	Environment
**YNB**		Environment
**5-FU**	10^{-4} M	Toxins
**5-FU**	10^{-6} M	Toxins
**6-azauracil**	1250 mg/ml	Toxins
**6-azauracil**	500 mg/ml	Toxins
**Arsenic**	0.5 mM	Toxins
**Arsenic**	0.05 mM	Toxins
**B-**	10 mM	Toxins

**mercaptoethanol**		
**Caffein**	2.25 mg/ml	Toxins
**Caffein**	1.5 mg/ml	Toxins
**Cycloheximide**	10^{-5} M	Toxins
**Cycloheximide**	10^{-6} M	Toxins
**DMSO**	8 %	Toxins
**DMSO**	6 %	Toxins
**DMSO**	4 %	Toxins
**DTT**	1.4 mM	Toxins
**Nystatin**	10^{-6} M	Toxins
**Nystatin**	5.10^{-7} M	Toxins
**Rapamycin**	10^{-7} M	Toxins
**Rapamycin**	10^{-8} M	Toxins
**SDS**	0.03 %	Toxins
**SDS**	0.01 %	Toxins
**SDS**	0.003 %	Toxins
